# Detection of Anti-Pentraxin-3 Autoantibodies in ANCA-Associated Vasculitis

**DOI:** 10.1371/journal.pone.0147091

**Published:** 2016-01-21

**Authors:** Amélie Simon, Jean-François Subra, Philippe Guilpain, Pascale Jeannin, Pascale Pignon, Simon Blanchard, Erwan Garo, Sébastien Jaillon, Alain Chevailler, Gilles Renier, Xavier Puéchal, Barbara Bottazzi, Alberto Mantovani, Yves Delneste, Jean-François Augusto

**Affiliations:** 1 Nephrology Department, University Hospital of Angers, Angers, France; 2 LUNAM University, Angers University, Cancer Research Center Nantes-Angers, Angers, France; Inserm, UMR 892, Angers, France; CNRS, UMR 6299, Angers, France; LabEx IGO, Angers, France; 3 Internal Medicine Department, University Hospital of Montpellier, Monptellier, France; 4 Immunology and Allergology Laboratory, University Hospital of Angers, Angers, France; 5 National Referral Center for Rare Systemic Autoimmune Diseases, Department of Internal Medicine, Hôpital Cochin, Assistance Publique-Hôpitaux de Paris, Paris, France; 6 Immunology and Inflammation Research Laboratory, Istituto Clinico Humanitas, Milano, Italy; Nippon Medical School Graduate School of Medicine, JAPAN

## Abstract

**Objectives:**

Pentraxin 3 (PTX3), in common with myeloperoxidase and proteinase 3, is stored in human neutrophil granules and is expressed on apoptotic neutrophil surface. We therefore investigated the presence of anti-PTX3 autoantibodies (aAbs) in the sera of antineutrophil cytoplasmic antibodies (ANCA)-associated vasculitis (AAV) patients.

**Methods:**

Presence of anti-PTX3 autoantibodies was analysed by a specific enzyme-linked immunosorbent assay in sera from 150 patients with microscopic polyangiitis (MPA), granulomatosis with polyangiitis (GPA), and eosinophilic granulomatosis with polyangiitis (EGPA), and in sera of 227 healthy subjects (HS), 40 systemic sclerosis (SSc) patients, and 25 giant cell arteritis patients (GCA). Using indirect immunofluorescence on fixed human neutrophils, we also analyzed the staining pattern associated with the presence of anti-PTX3 aAbs.

**Results:**

Anti-PTX3 aAbs were detected in 56 of 150 (37.3%) of the AAV patients (versus 12 of 227 (5.3%) of HS, p<0.001) and, interestingly, in 7 of 14 MPO and PR3 ANCA negative AAV patients. Moreover, by indirect immunofluorescence on fixed neutrophils, anti-PTX3 aAbs gave rise to a specific cytoplasmic fluorescence pattern distinct from the classical cytoplasmic (c-ANCA), perinuclear (p-ANCA), and atypical (a-ANCA) pattern. Anti-PTX3 aAbs levels were higher in patients with active AAV as compared to patients with inactive disease.

**Conclusion:**

Our work suggests that PTX3 is as a novel ANCA antigen. Anti-PTX3 aAbs appear thus as a promising novel biomarker in the diagnosis of AAV, including in patients without detectable MPO and PR3 ANCA.

## Introduction

Microscopic polyangiitis (MPA), granulomatosis with polyangeitis (GPA), and eosinophilic granulomatosis with polyangeitis (EGPA) are vasculitides characterized by necrotizing inflammation of small to medium-sized vessels. They are usually associated with serum positivity for anti-neutrophil cytoplasmic antibodies (ANCAs) [1, 2]. In most cases, ANCAs are directed against two constituents of neutrophil primary granules and monocyte lysosomes: myeloperoxydase (MPO) or proteinase 3 (PR3). In ANCA-associated vasculitis (AAV) pathophysiology, neutrophils appear both as targets and effectors of the auto-immune process [3]. Diagnosis of MPA, GPA, and EGPA is based on identification of pauci-immune small-vessels vasculitis at pathology. However, detection of MPO or PR3 ANCAs are of major interest for the prompt diagnosis and the follow up of AAV [4]. Using indirect immunofluorescence (IIF) on fixed neutrophils and ELISA, ANCAs are detected in most of MPA and GPA patients, but in less than half of the patients with EGPA [5]. In the absence of detectable ANCA, the diagnosis of "seronegative" AAV is thus difficult to assess. While some authors have reported that MPO or PR3 ANCA titers may correlate with disease activity [6], others have demonstrated that ANCA titers are not valuable to guide treatment. Therefore, it seems crucial to identify new reliable biomarkers, particularly in seronegative ANCA disease, for diagnosis and follow-up of the disease [7].

Pentraxins are soluble pattern recognition receptors belonging to the humoral arm of the innate immune system. They are involved in the clearance of non-self (pathogens) and modified-self (apoptotic cells) [8]. The pentraxin family is composed of two structural classes: short and long pentraxins. The first class includes the acute phase proteins C-reactive protein (CRP) and serum amyloid P component (SAP), and the second class includes the long pentraxins PTX3. The prototypic long pentraxin PTX3 is a 381 amino-acids long protein (45 kDa) consisting of a 203 amino-acids C-terminal pentraxin-like domain associated with a 178 amino-acids N-terminal portion, unrelated to other known proteins [9]. Unlike short pentraxins produced by the liver in response to IL-6, PTX3 is produced by various cell types including endothelial cells [10], fibroblasts, myeloid cells [11], and epithelial cells [12] in response to pro-inflammatory mediators (IL-1β, TNFα) and TLR agonists. PTX3 acts as an opsonin and protects the host against infections by various pathogens such as *aspergillus fumigatus* [13].

The presence of preformed PTX3 in neutrophil granules [14], similar to MPO and PR3 [15, 16], and the detection of circulating anti-PTX3 aAbs in other autoimmune disease such as systemic lupus erythematosus [17, 18], lead us to investigate whether anti-PTX3 aAbs could be detected in the sera of AAV patients.

We report here that 40% of AAV patients exhibit anti-PTX3 aAbs. Furthermore, anti-PTX3 aAbs can be detected in 50% of patients with AAV without both MPO and PR3 ANCAs.

## Patients and Methods

### Patient Serums

161 serums from 150 AAV patients were obtained from the Immunology laboratories of the University Hospital of Angers (France), from the Le Mans General Hospital (France) and from The National Referral Center for Necrotizing Vasculitis and Systemic Sclerosis (Cochin University Hospital, Paris, France). Among the 150 patients, 93 patients had active AAV and 57 had remittent disease. Among patients with active disease, 70 patients were sampled at AAV diagnosis and 23 patients were sampled at AAV relapse. For eleven patients with active disease, a second sample was available 3 to 12 month later at remission. Written informed consent was obtained from each donor. The study protocol was in agreement with the ethics committee of the Angers University Hospital (2011–06).

Classification of AAV (GPA, MPA and EGPA) was determined according to the European Medicines Agency (EMEA) vasculitis classification algorithm [19]. Among the cohort, 74 patients were classified as having MPA, 54 as having GPA, and 22 as having EGPA. Serums were aliquoted and kept frozen to avoid repeated thawing-freezing. Serums from 227 healthy subjects (HS) (Blood Collection Center, Angers, France; agreement ANG 2003–2), 40 systemic sclerosis (SSc) patients, and 25 giant cell arteritis (GCA) patients were used as controls.

Exhaustive clinical data were available for 60, 42 and 13 patients with MPA, GPA, and EGPA, respectively. Disease activity was determined using the Birmingham vasculitis activity score (BVAS 2003) according to the European League Against Rheumatism (EULAR) recommendations [20].

In a previous study, sera from healthy subjects, from 36 systemic lupus erythematosus patients and from 40 patients with rheumatoid arthritis were tested [17].

### ANCA Detection

All sera were tested for the presence of ANCAs by ELISA Kits from Euroimmun according to the manufacturer recommendations (Anti-PR3 IgG ELISA, #EA 1201-9601-2 G; anti-MPO IgG ELISA, #EA 1211–9601 G; and ANCA-Profile ELISA IgG, #EA 1200-1208-1 G; EuroImmun, Lübeck, Germany). Briefly, 96 wells-plates coated with PR3, MPO, lactoferrin, elastase, BPI, or cathepsin G were incubated with 100 μl of diluted sera for 30 minutes. After washing, 100 μl of HRP-labeled rabbit anti-human IgG was incubated for 30 minutes. Bound aAbs were detected with TMB. Samples were considered positives if OD was higher than cut-off value provided by manufacturer.

### Detection of Anti-Pentraxin aAbs by ELISA

96-wells plates (Nunc, Roskilde, Denmark) were either uncoated or coated overnight at 4°C with 50 μL of 10 μg/ml pentraxin in 50 nM carbonate-bicarbonate buffer (pH = 9.6) (Sigma Aldrich, Saint-Louis, MO). Recombinant human PTX3 was produced and purified as previously described [21]. CRP and SAP were purchased from Millipore (Billerica, MA) and Calbiochem (Merck, Darmstadt, Germany), respectively. The 1–141 N-terminal peptide of PTX3 was chemically synthesized (Synprosis, Marseille, France) and used at 5 μg/ml. After saturation with 200 μL 1% bovine serum albumin (Euromedex, Souffelweyersheim, France) in endotoxin-free PBS (Lonza, Verviers, Belgium), 100 μL of 1:400 diluted serum in PBS, 0.5% BSA, 0.05% Tween 20, were added and incubated for 2 hours at 37°C. Plates were then washed 3 times with 200 μL PBS, 0.05% Tween 20, and incubated at 37°C for 1 hour with biotin-labeled goat anti-human heavy chain immunoglobulin antibody (Jackson Immunoresearch, West Grove, PA, #109-066-088) diluted in PBS, 0.5% BSA, 0.05% Tween 20. After washing, streptavidin-HRP (R&D Systems, Minneapolis, MN) was added and incubated 1 hour at 37°C. Plates were washed and bound Abs were detected using the 3,3,5,5’-tetramethyl-benzidin (TMB) substrate. Optical density (OD) at λ = 450 nm minus λ = 570 nm was measured in a spectrophotometer. Result for each sample is expressed as the net OD value obtained after subtraction of the OD obtained with the antigen-free well to the OD obtained with pentraxin-coated well. The OD values were determined as described above.

Cut-off for positivity of anti-PTX3, anti-CRP and anti-SAP aAbs was determined as OD value higher than the mean OD value + 2 standard deviations (SD) in healthy subject sera [17]. Samples were run in duplicate and intra-assay variation was less than 5%.

### Detection of Anti-Pentraxin aAbs by Indirect Immunofluorescence

Wells containing ethanol-, formaldehyde- and methanol-fixed granulocytes and HEp-2 cells (EUROPLUS™ Granulocyte Mosaic 23; Euroimmun) were incubated with 2.5 μg/ml rabbit anti-human PTX3 polyclonal Abs in PBS containing 0.5% Tween 20, for 30 minutes, at room temperature. After washing, 20 μg/ml FITC-labeled goat anti-rabbit immunoglogulin Ab (Invitrogen, Carlsbad, CA) was incubated for 30 minutes at room temperature. After washing and inclusion in glycerol, fluorescence was analyzed by microscopy (Colibri; Carl Zeiss, Göttingen, Germany). The same procedure was used with diluted human sera using a DyeLight488-labeled anti-human immunoglobulin as secondary antibody (Euroimmun). Sera giving rise to cytoplasmic (c-ANCA), perinuclear (p-ANCA), and atypical (a-ANCA) fluorescence staining patterns (from the collection of the Immunology and Allergology Department, University Hospital of Angers, France) were used as controls.

### Statistical Analysis

Results are expressed as median OD value and range. Statistical analysis were performed using Prism 5 software (GraphPad software Inc.). Mann-Whitney U test was used to compare variables between groups of healthy subjects and vasculitis patients and Kruskall-Wallis test was used to compare variables when more than two groups were present. The χ^2^ test (or Fisher exact test) was used to compare categorical variables between groups. Wilcoxon tests were performed to compare paired data and correlations were assessed using Spearman’s correlation test. Differences were considered significant when p<0.05.

## Results

### Detection of Anti-PTX3 Antibodies in AAV Patients

**Fig 1:**
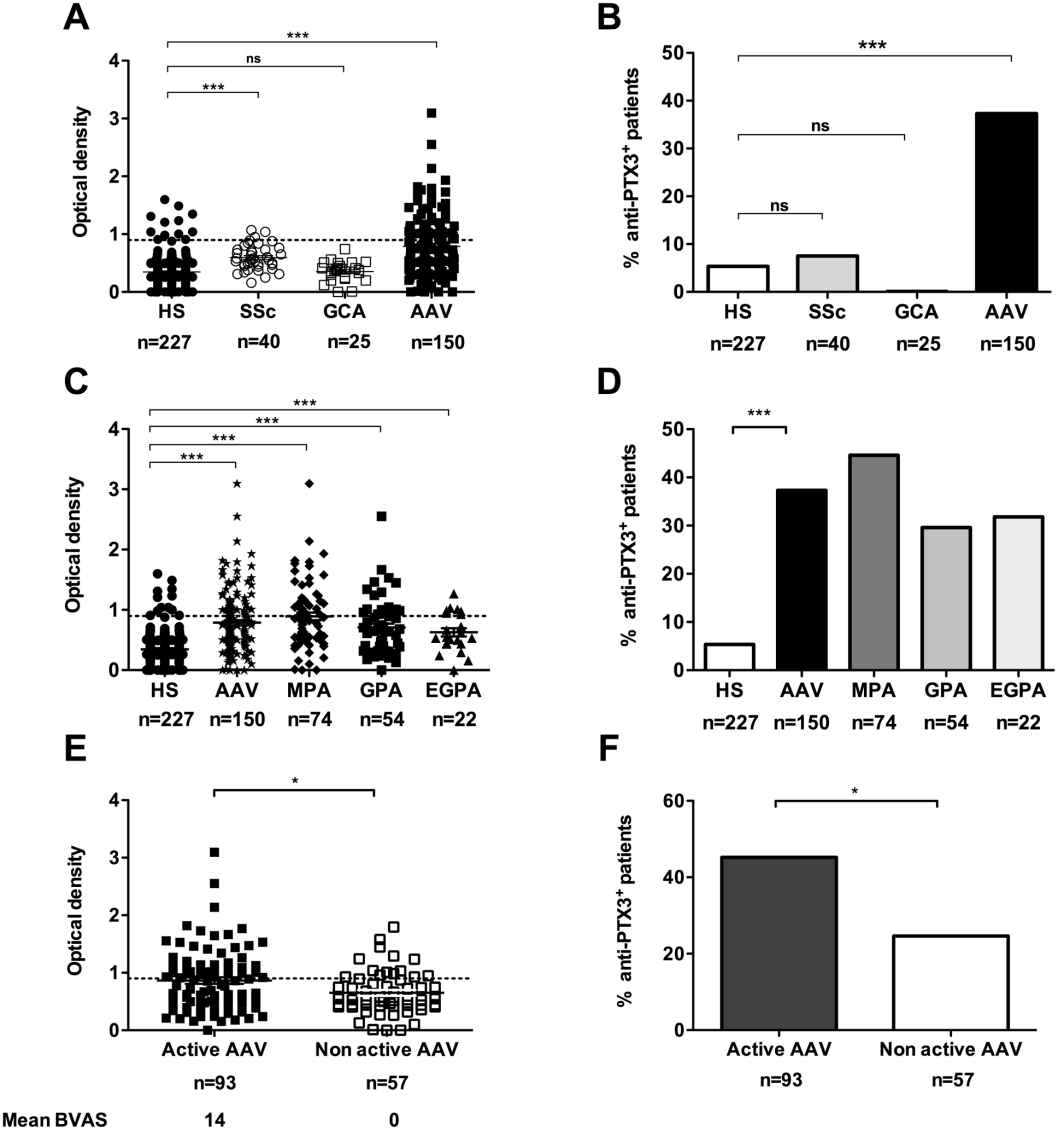
Prevalence of anti-PTX3 aAbs in AAV patients. **A,** Anti-PTX3 aAbs were analysed by ELISA in 150 AAV patients with MPA, GPA, or EGPA, 40 patients with SSc, 25 patients with GCA and of 227 sera from healthy subjects (HS). **B**, Prevalence of anti-PTX3 aAbs in AAV, SSc, GCA patients and HS. **C**, Anti-PTX3 aAbs according to AAV subtypes (MPA, GPA or EGPA). **D**, Prevalence of anti-PTX3 aAbs according to the AAV subtypes. **E**, Anti-PTX3 aAbs in AAV patients according to disease status (active or non active disease). F, Prevalence of anti-PTX3 aAbs according to AAV status. **A, C, E**, Results are expressed in OD values. Dotted line corresponds to mean + 2 SD titres of anti-PTX3 aAbs in HS sera; full line corresponds to the mean OD in each group of patients. B, D, F, Results are expressed in percentage. ***p<0,001; *p<0.05; ns, not significant.

We investigated by ELISA the presence of anti-PTX3 aAbs in AAV patients (Table 1), SSc patients, GCA patients and HS. We detected anti-PTX3 aAbs in 56 of 150 (37.3%) AAV patients (Fig 1A and 1B). Anti-PTX3 aAbs were detected in 3 of 40 (7.5%) of SSc patients, in none of the 25 GCA patients and in 12 of 227 HS (Fig 1A). Anti-PTX3 aAbs were detected in 44.6%, 29.6%, and 31.8% of patients with MPA, GPA, and EGPA, respectively (median OD = 0.82 [0.01–3.09], n = 75; 0.65 [0.01–2.55], n = 54; and 0.54 [0.01–1.27], n = 22; respectively) (Fig 1C and 1D). Anti-PTX3 aAbs were detected with a higher prevalence in AAV patients with active disease as compared to patients with non-active disease (45,2% and 24,6% respectively, p = 0;011, Fig 1E and 1F).

**Table 1:** Demographic and clinical features of AAV patients. BVAS, Birmingham vasculitis activity score; EGPA, eosinophilic granulomatosis with polyangiitis; ENT, Ear nose throat; ELISA, Enzyme-linked immunosorbent assay; GPA, granulomatosis with polyangiitis; MPA, Micropolyangiitis.

	EGPA	GPA	MPA
**Total patients**			
Number of patients	22	54	74
Anti-MPO+ and/or anti-PR3+ (ELISA)	13.6%	85.1%	79.7%
Active/non-active disease	9/13	34/20	50/24
**Patients with exhaustive clinical data**			
Number of patients, n	13	425.2	60
Male/Female, n number)	9/4	20/22	32/28
Mean age at diagnosis, years	47.3	52.7	60.9
Active / non-active disease, n (mean BVAS)	8 (13) / 5 (0)	34 (16) / 8 (0)	50 (16) / 10 (0)
Anti-MPO^+^ and/or anti-PR3^+^ (ELISA)	23.1%	76.2%	70%
**Clinical manifestations (%)**			
General signs	76.9	76.2	76.6
Joints	23.1	52.4	53.3
Cutaneous	61.5	45.2	41.7
Mucous membranes/ Eyes	69.2	30.9	10
ENT	0	69	13.3
Chest	84.6	64.3	41.7
Cardiovascular	38.5	2.4	11.7
Abdominal	30.8	7.1	15
Renal	15.4	59.5	68.3
Nervous system	53.8	23.8	38.3

**Fig 2:**
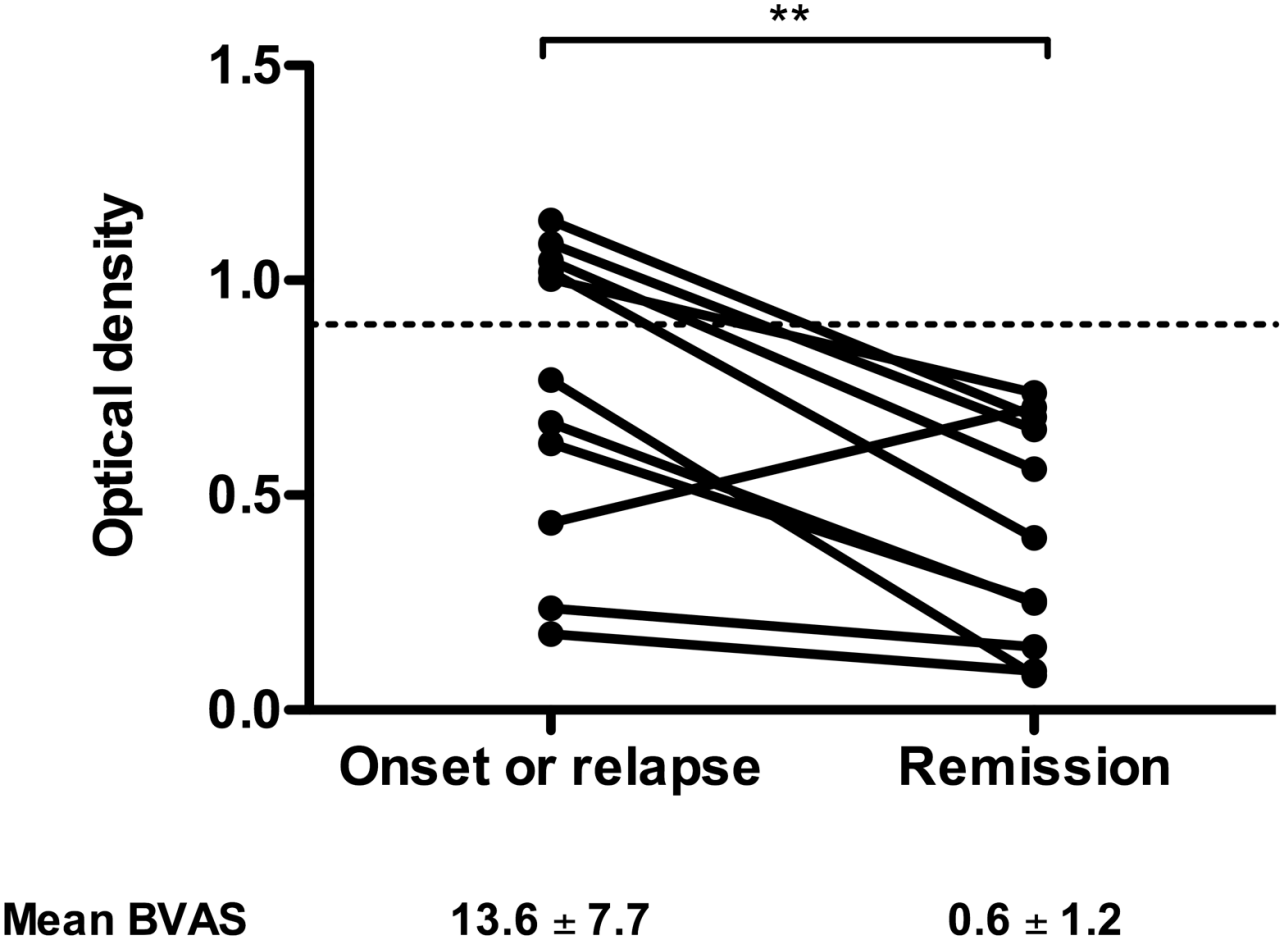
Longitudinal analysis of anti-PTX3 aAbs. Anti-PTX3 aAbs were analysed by ELISA in 14 patients with active disease (onset or relapse) and at remission. Results are expressed in OD values. Dotted line corresponds to mean + 2 SD titres of anti-PTX3 aAbs in HS sera; full line corresponds to the mean OD in each group of patients. Each line represents one patient. *p<0.05.

In eleven AAV patients, serums were available at disease onset and later (3 to 12 months) at remission. We observed a significant decrease in anti-PTX3 level after disease remission (median OD = 0.77 [0.18–1.14], 0.40 [0.08–0.74], p = 0.0025) (Fig 2). We also detected anti-PTX3 aAbs in 36.0% of MPO ANCA positive sera and in 35.1% of PR3 ANCA positive sera (data not shown). No association was found between clinical involvement of AAV and anti-PTX3 aAb positivity (data not shown).

### Prevalence of Anti-PTX3 Antibodies in ANCA-Negative AAV

In this cohort of MPA, GPA and EGPA patients, we investigated whether patients defined as "ANCA negative" may have anti-PTX3 aAbs. Among the 150 AAV patients, 14 patients (6 EGPA, 6 MPA, and 2 GPA), sampled at the time of diagnosis (before and during the first month of treatment), were negative for both MPO and PR3 ANCAs and also for anti-lactoferrin, -BPI, -elastase, and -cathepsin G aAbs. Interestingly, in these patients, anti-PTX3 aAbs were detected in 7/14 patients (5 MPA and 2 EGPA).

### Anti-CRP and Anti-SAP Antibodies in AAV

**Fig 3:**
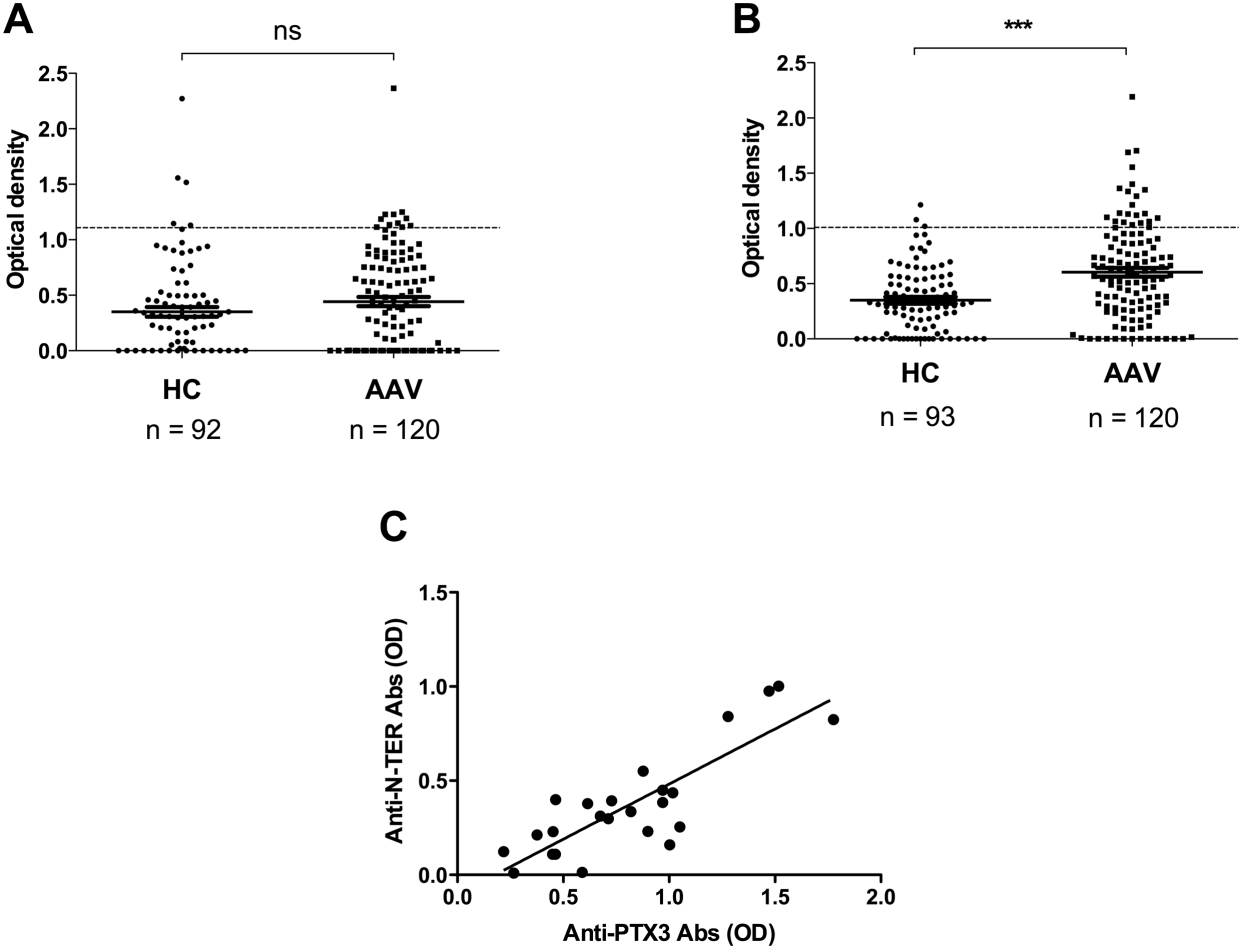
Anti-pentraxin aAbs in AAV patients and specificity of anti-PTX3 aAbs. The presence of anti-CRP **(A)**, anti-SAP **(B)** aAbs were investigated by ELISA in 120 AAV sera (randomly selected; anti-PTX3 aAbs were detected in 53 sera out of 120 [44.2%]) and in healthy subjects (HS). **A&B**, Dotted lines correspond to mean + 2 SD titers of anti-CRP and -SAP aAbs in HS sera. Full lines correspond to the mean reactivity + SEM of sera in each group. ***p<0.001. **C**, The titers of anti-PTX3 (x-axis) and anti-PTX3 N-terminal domain (y-axis) were both determined in 24 AAV sera (randomly selected). Association was analysed using Spearman’s rank correlation.

Anti-CRP and -SAP aAbs have been reported in patients with systemic lupus erythematosus [22, 23]. We therefore evaluated whether circulating anti-CRP and/or -SAP aAbs were also detectable in AAV. Anti-PTX3, -CRP, and -SAP aAbs were evaluated by ELISA in 120 randomly selected sera of AAV patients. As reported by Tan [24], anti-CRP aAb titer was not significantly different between AAV patients and HS (median OD = 0.39 [0–2.4] vs 0.26 [0–2.3], respectively) (Fig 3A). Moreover, although anti-SAP aAb titers were higher in AAV patients than healthy subjects (median OD = 0.57 [0–2.2] vs 0.31 [0–1.21]; p<0.001), only 17.5% of the AAV sera were positive for anti-SAP aAbs (Fig 3B) compared with 44.2% for anti-PTX3 aAbs; in these randomly selected sera. Among the anti-SAP+ sera, two of them were ANCA negative and both were positive for anti-PTX3 aAbs.

### Cross Reactivity of the Anti-PTX3 Antibodies

The pentraxins PTX3, CRP, and SAP share 17% amino-acid homology in the C-terminal domain. We therefore had to exclude that PTX3 immunoreactivity did not result from recognition of PTX3 by anti-short pentraxin aAbs. First, among 53 anti-PTX3 aAb positive sera, 40 (75.5%) were negative for both anti-CRP and -SAP aAbs (Table 2). Second, titers of aAbs directed against the N-terminal domain of PTX3 (absent in CRP and SAP) correlated with anti-PTX3 aAb titers (r = 0.75, p<0.001) (Fig 3C). These results suggest that most of the anti-PTX3 aAbs detected bind selectively to PTX3 and not to CRP or SAP.

**Table 2:** Presence of anti-CRP, -SAP, and -PTX3 aAbs in 120 sera of AAV patients.

		Anti-CRP aAbs^+^ (%)	Anti-SAP aAbs^+^ (%)	Anti-CRP aAbs^+^ anti-SAP aAbs^+^ (%)	Anti-CRP aAbs^-^ anti-SAP aAbs^-^ (%)
Anti-PTX3 aAbs^+^	53	4 (7.5)	6 (11.3)	3 (5.7)	40 (75.5)
Anti-PTX3 aAbs^-^	67	2 (3.0)	11 (16.4)	1 (1.5)	53 (79.1)
Total	120	6 (5.0)	17 (14.2)	4 (3.3)	93 (77.5)

### Fluorescence Pattern of Anti-PTX3 Antibodies on Fixed-Neutrophils

**Fig 4:**
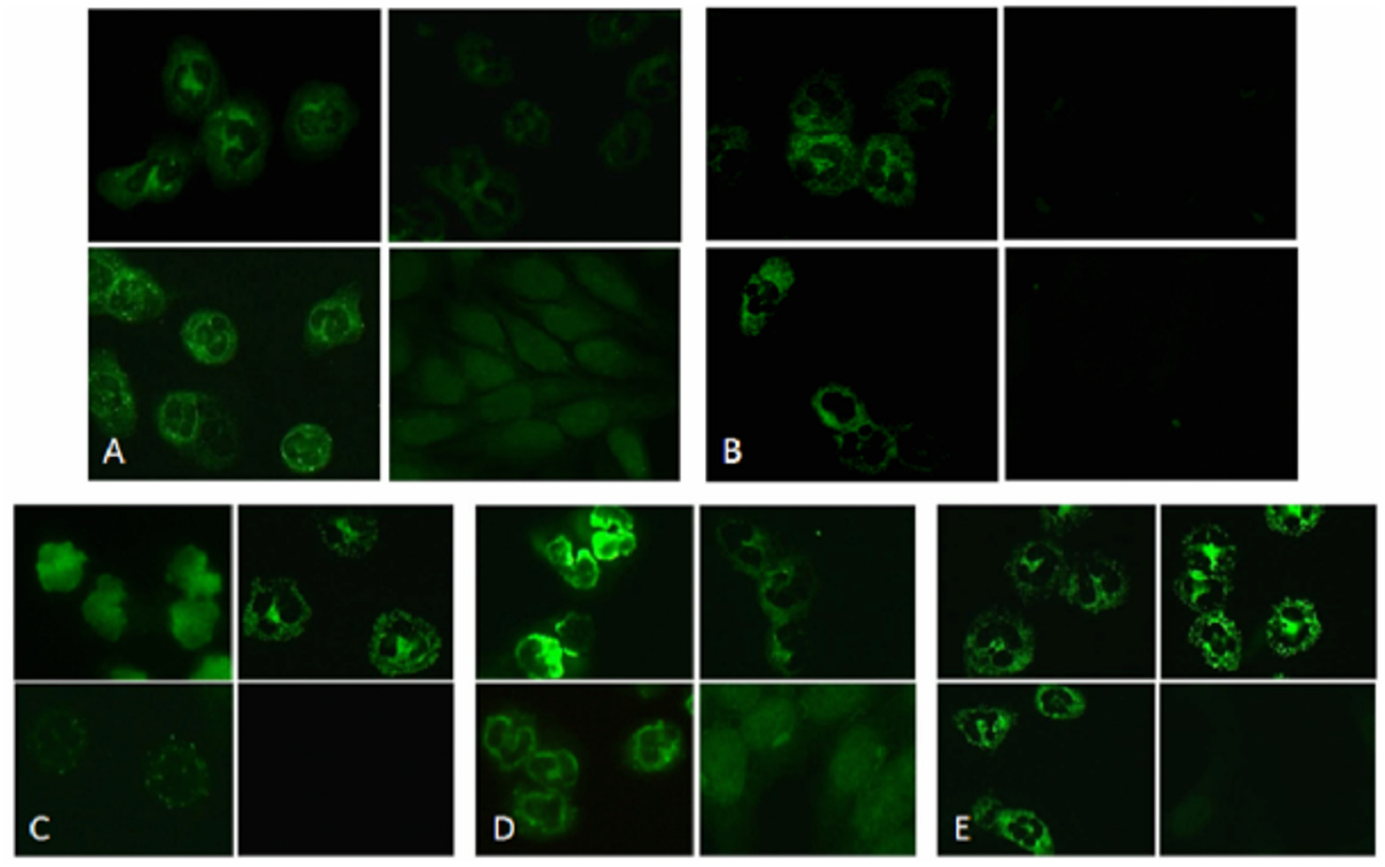
Immunofluorescence staining of neutrophils by anti-PTX3 Abs. Slides containing 4 biochips as substrate ethanol-fixed (upper left), formol-aceton fixed neutrophils (upper right) methanol-fixed neutrophils (lower left) and HEp2 cells (lower right) were incubated with **A**, a serum from a patient with anti-PTX3 aAbs and neither MPO, PR3, BPI, lactoferrine, elastase nor cathepsin G ANCA, **B**, an anti-PTX3 polyclonal antibody or **C-E**, control sera for p-ANCA (**C**), a-ANCA (**D)**, c-ANCA (**E**). **A, C-E**, results are representative of the results obtained with one out of four sera. B, Results are representative of one out of three experiments.

Indirect immunofluorescence (IIF) on human fixed-neutrophils remains the gold standard method for ANCA detection. Given that PTX3 is stored in neutrophil granules [14], we therefore evaluated whether the presence of anti-PTX3 aAbs could be associated with a specific immunofluorescence staining pattern. Sera from 7 patients positives for anti-PTX3 aAbs and negative for MPO, PR3, BPI, lactoferrin, cathepsin G and elastase ANCA were incubated with human neutrophils fixed in methanol, ethanol or formol-acetone. A staining of small cytoplasmic granules was observed in methanol- and ethanol-fixed neutrophils in 4 out of 7 patients (Fig 4A). The 3 other sera were IIF negative at the tested dilution (1/20). A similar fluorescent pattern was observed with a rabbit anti-human PTX3 polyclonal Ab, used as a positive control (Fig 4B). Fluorescence aspect observed with the anti-PTX3 positive sera was different from the classical p- and a-ANCA (Fig 4C and 4D, respectively) pattern due to the absence of homogenous perinuclear staining on ethanol-fixed neutrophils and closer to c-ANCA (Fig 4E). Nevertheless, the fluorescence aspect with anti-PTX3 positive sera differed from the c-ANCA pattern, as the cytoplasmic granules stained were smaller, visible in methanol and ethanol-fixed neutrophils, and less detectable in formol-fixed neutrophils (Fig 4E). We called this typical staining sc-ANCA for small cytoplasmic ANCA.

## Discussion

We report here for the first time the presence of anti-PTX3 aAbs in AAV. Moreover, we observed that anti-PTX3 aAbs were associated with a specific IIF pattern on human fixed neutrophils suggesting that PTX3 is a novel ANCA antigen. Interestingly, half of the patients without MPO and PR3 ANCAs (7/14) at diagnosis displayed anti-PTX3 aAbs in their sera. These results suggest that anti-PTX3 aAbs determination may be of potential interest as a diagnostic or a follow-up biomarker in AAV patients.

Interestingly, serum PTX3 levels have been found increased in AAV patients as compared to healthy subjects and to be higher in patients with active AAV [25]. PTX3 is secreted by a wide variety of cells including endothelial cells and neutrophils [11]. In common with MPO and PR3, PTX3 is stored in human neutrophil granules. Thus, we analysed the presence of anti-PTX3 aAbs in AAV. We found anti-PTX3 aAbs in nearly 40% of the patients with MPA, GPA, or EGPA. In contrast, anti-PTX3 aAbs were not detected in the sera of our control group of patients with other inflammatory diseases i.e. psoriasic arthritis, rheumatoid arthritis, Sjogren’s syndrome, or systemic sclerosis [17, 18], thereby suggesting that the presence of anti-PTX3 aAbs may be limited to some autoimmune disorders (i.e. systemic lupus and vasculitis). We did not observe any difference of anti-PTX3 aAbs prevalence between GPA, MPA and EGPA subgroups, showing that anti-PTX3 aAbs are not helpful for distinguishing AAV entities. We also did not observe anti-PTX3 aAbs in GCA patients, suggesting that anti-PTX3 aAbs may be limited to some vasculitis subtypes. Interestingly, anti-PTX3 aAbs were detected in roughly 35% of both MPO- and PR3-ANCA positives patients. This observation is in line with previous data showing a high prevalence of dual ANCA positivity to both MPO/PR3 ANCAs and minor ANCA antigens in AAV patients [26].

Up to 20% of the patients with confirmed diagnosis of GPA or MPA and 40% of patients with EGPA are MPO and PR3 ANCA negative. Roth et al recently reported that serum ceruloplasmin has the property to bind to MPO, masking some MPO epitopes and rendering MPO ANCA undetectable by standard methods [27]. This mechanism may explain part of the ANCA negative patients. Our results show that anti-PTX3 aAbs are present in half of AAV patients without MPO or PR3 ANCAs at diagnosis. Thus, anti-PTX3 aAbs may represent a biomarker to assess AAV diagnosis of PR3/MPO ANCA negatives patients. These data also reinforce the interest in investigating anti-PTX3 aAbs, together with MPO and PR3 ANCAs, in patients with suspected AAV. However, these results need cautious interpretation and confirmation given the small subgroup of ANCA negative patients of our study.

Furthermore, AAV is a relapsing and remitting disease for which biological markers are needed to evaluate disease activity and to predict relapses. We observed that anti-PTX3 aAb reactivity and prevalence was higher in patients with active AAV as compared to patients with remittent disease. Moreover, in a longitudinal analysis including 11 patients, anti-PTX3 aAb reactivity decreased significantly after remission. These observations suggest that anti-PTX3 aAbs may be useful to monitor AAV activity, even though additional studies are needed to specifically investigate this point.

As PTX3 shares 17% amino acid homology with short pentraxins, it was important to exclude that aAbs against PTX3 could be mainly anti-CRP or anti-SAP aAbs cross-reacting with PTX3. Our results show that anti-CRP and anti-SAP aAbs are not significantly detected in AAV. Moreover, CRP and SAP are not detected in neutrophil cells.

The sera of AAV patients with anti-PTX3 aAbs show a IIF specific pattern in ethanol and methanol-fixed neutrophils but not in formol-fixed neutrophils, with smaller fluorescent granules than c-ANCA. The cytoplasmic and the usual perinuclear pattern are commonly associated with Abs directed to PR3 and MPO, respectively. These preformed proteins colocalize in primary azurophilic granules of neutrophils [15, 16]. In contrast, PTX3 is stored in secondary, lactoferrin-positive granules. These different localizations may explain the specific pattern observed with anti-PTX3 aAbs.

In vitro studies have suggested a pathogenic role for ANCA in the vascular damages linked to MPA, GPA, and EGPA. MPO and PR3 translocate at the surface of neutrophils stimulated by pro-inflammatory cytokines, allowing the interaction of ANCA with their antigenic targets. Binding of ANCA induces neutrophil activation, resulting in increased adhesion, migration to endothelium and release of proteolytic granules enzymes, proinflammatory cytokines, generation of respiratory burst and eventually endothelial cell damage [28, 29]. Animal models provide conclusive evidence that anti-MPO aAbs induce small vessel vasculitis. In contrast, the pathogenicity of PR3 ANCA in vivo is less clear [30–32]. To date, the pathogenic role of anti-PTX3 aAbs remains to be determined. We have observed that preformed PTX3 stored in neutrophil granules is translocated at the surface of late apoptotic neutrophils acting as an “eat-me” molecule [33]. The clearance of apoptotic cells must be immunologically silent to maintain self-tolerance [34]. We can hypothesize that anti-PTX3 aAbs opsonise apoptotic neutrophils (that express cell surface PTX3), favour their uptake by phagocytes in an immunostimulatory context, and thereby enhance their immunogenicity. Supporting this hypothesis, apoptotic neutrophils also express PR3 and MPO at their surface and ANCA interaction with MPO and PR3 induce their phagocytosis associated with the production of pro-inflammatory cytokines [35, 36]. PTX3 is also highly expressed by endothelial cells, especially in inflammatory conditions [25, 37]. Thus, another way of pathogenicity of anti-PTX3 aAbs may be endothelial cell injury.

Thus, anti-PTX3, like MPO and PR3 ANCA, may participate to vasculitis pathophysiology, favouring primed neutrophil degranulation and/or clearance of apoptotic neutrophil in an immunogenic context. Additional experiments are in progress to determine whether anti-PTX3 aAbs modulate the immunogenicity of apoptotic cells and thereby contribute to the maintenance of the disease.

Recent data identified neutrophil extracellular traps (NETs) as potential players in AAV pathophysiology [38]. “NETosis” is a specific type of neutrophil cell death characterized by the release of chromatin fibers “decorated” with neutrophil cytoplasmic proteins. In vitro, ANCAs induce NETs release by primed neutrophils and Kessenbrock et *al* demonstrated the presence of MPO and PR3 in NETs [38]. Supporting the implication of NETosis in AAV pathophysiology, NETs constituents have been identified in kidney biopsies of AAV patients. Moreover, recent data also demonstrated that NETs are able to transfer neutrophil autoantigens, such as MPO, elastase or PR3 to dendritic cells, and trigger autoimmunity [39]. Interestingly, we recently reported that PTX3 was also localized in NETs [14]. Moreover, in a previous work, we demonstrated that anti-PTX3 aAbs prevalence was low in rheumatoid arthritis patients, while detected in 50% of patients with SLE, a disease also associated with excessive NETosis [17, 40]. Taken together, these data suggest that anti-PTX3 aAbs may have an implication in AAV pathophysiology and give an hypothesis on the mechanism of PTX3 tolerance breakdown.

In conclusion, our work suggests that PTX3 is a novel ANCA antigen. We observed a high prevalence of anti-PTX3 aAbs in AAV patients. Interestingly, anti-PTX3 aAbs were detected in a proportion of ANCA-negative patients. Altogether, these data suggest that anti-PTX3 aAbs may be interesting as a biomarker. Several lines of evidence suggest that anti-PTX3 aAbs may be involved in the pathophysiology of AAV.

## Abbreviations

aAbs: autoantibodies
a-ANCA: atypical fluorescence pattern
ANCA: Antineutrophil cytoplasmic antibodies
AAV: ANCA-associated vasculitis
c-ANCA: cytoplasmic fluorescence pattern
CRP: C-reactive protein
EGPA: eosinophilic granulomatosis with polyangiitis
GCA: giant cell arteritis
GPA: granulomatosis with polyangiitis
MPA: microscopic polyangiitis
MPO: myeloperoxydase
p-ANCA: perinuclear fluorescence pattern
PTX3: pentraxin 3
PR3: proteinase 3
SAP: serum amyloid P
SSc: systemic sclerosis
